# Development and validation of the Perceived Benefits of Team-Interaction Training Questionnaire (PBTITQ) among undergraduates

**DOI:** 10.1186/s12909-023-04810-3

**Published:** 2023-11-07

**Authors:** Ming Chen, Hefang Chen, Yifan Wu, Ruijun Yang, Chaowei Guo, Meizhen Zhao, Chaoli Xin, Shuang Zang

**Affiliations:** 1 Teaching and Research Department of P.E, China Medical University, No.77 Puhe Road, Shenyang North New Area, Shenyang, 110122 China; 2 Department of Nursing, the First Hospital of China Medical University, No.155 Nanjing North Street, Heping District, Shenyang, 110000 China; 3 Department of Basic Nursing, School of Nursing, Jilin University, No. 965 Xinjiang Street, Changchun, 130021 China; 4 School of Nursing, China Medical University, No.77 Puhe Road, Shenyang North New Area, Shenyang, 110122 China; 5 Department of Nursing, Tongji Hospital, Tongji Medical University, Huazhong University of Science and Technology, No.1095, Jiefang Avenue, Qiaokou District, Wuhan, 430030 China; 6 School of Management, Guizhou Business School, 94 Xihu Road, Nanming District, Guiyang, 550014 China

**Keywords:** Team-interaction, Perceived benefits, Training, Validity, Undergraduates

## Abstract

**Background:**

The effectiveness of team-interaction training has been proven. However, there is a lack of objective and accurate evaluation tools for the impact and benefits of team-interaction training on participants. This study aims to develop and validate a tool for exploring undergraduates’ perception of benefits in team-interaction. It can further insight into the perceived benefits of team-interaction training for undergraduates and evaluates the effectiveness of the course, and provides a reference point for the development of university team-interaction training courses.

**Methods:**

This study was conducted in three stages. Phase 1 consisted of item generation: A theoretical framework was crafted based on social cognitive theory, self-efficacy theory, and sports performance models. Fifty-two items were generated based on the theoretical framework, participant interviews, and literature review. After Delphi consultation and pilot tests, 39 items moved on to Phase 2. Phase 2 consisted of forming a preliminary questionnaire: the contents to be included were selected through item analysis and exploratory factor analysis (EFA). A total of 40 classes were selected for EFA. After EFA, a three-factor structure with 25 items was formed. The third stage tested psychometric properties through confirmatory factor analysis (CFA), test-retest reliability, criterion-related validity, and internal consistency.

**Results:**

The final PBTITQ consisted of 23 items, each rated from “1” (fully disagree) to “5” (fully agree). EFA and CFA supported the three-factor structure of PBTITQ, which included Cohesion, Communication, and Efficiency. The Cronbach’s alpha of the PBTITQ was 0.90, the test-retest reliability was 0.88, and the split-half reliability was 0.81. PBTITQ significantly correlated with the GEQ (*r* = 0.808, *p* < 0.05) and the TDM (*r* = 0.796, *p *< 0.05).

**Conclusion:**

The PBTITQ is an effective tool for assessing the perceived benefits of team-interaction training among undergraduates.

**Supplementary Information:**

The online version contains supplementary material available at 10.1186/s12909-023-04810-3.

## Background

Team-interaction is a dynamic and changing sequence of social actions between individuals, including monitoring, coordinating, and communicating, which are regarded together as the dominant methods of teamwork [1]. Previous studies have shown that team-based cooperation at work significantly improves productivity, increases team performance, and reduces the occurrence of work errors [2–6].

Currently, in a significant number of recruitments, companies often list team-interaction skills as an important requirement [7]. Team-interaction seems to be becoming an essential competency that is crucial for undergraduates. However, traditional academic culture does not take into account the importance of team-interaction [8]. Traditional curricula do not seem to address the skill needs of the workplace. As a result, some universities have begun to incorporate team-interaction into their syllabi [9]. Research has shown that team-interaction training for undergraduates facilitates the exchange of knowledge and ideas as well as practical skills [10]. This not only facilitates their rapid integration into the job market, but also improves their academic performance and supports their development throughout their academic and professional careers. Based on the emergence of such training, there is a need for a suitable evaluation tool to assess the effectiveness of team-interaction training.

Questionnaires are tools for the quantitative evaluation of the effectiveness of training. A number of questionnaires have been developed in the past to assess the effectiveness of team-interaction training. For example, the Group Environment Questionnaire (GEQ) is designed to measure the perceived benefit of student cohesion [11]. The TeamSTEPPS Team Perception Questionnaire is intended to assess individuals’ perception of team-interaction skills and behaviours [12]. The Team Development Measure (TDM) is designed to evaluate the efficacy of the team and the team-based problems that prohibit this development [13]. Although researchers have tried to develop some assessment tools on team interaction training from different perspectives, there is still a lack of suitable questionnaires to quantitatively measure undergraduate perception of benefits of team interaction.

Some scholars have indicated that the choice to engage in social behaviour, such as participating in team interaction, should be analysed in terms of benefits, as they are subjectively perceived by the participants [14]. Findings from university cooperative learning showed that perceived benefit mediates the effects of external conditions on learning and provides valuable insights [2]. Therefore, perceived benefits play an imperative role as the key link in evaluating team-interaction training.

The purpose of this study was to develop and validate an instrument to assess the perceived benefits of team-interaction training for undergraduate. It can further insight into the perceived benefits of team-interaction training for undergraduates and evaluates the effectiveness of the course, and provides a reference point for the development of university team-interaction training courses.

## Methods

**Fig. 1:**
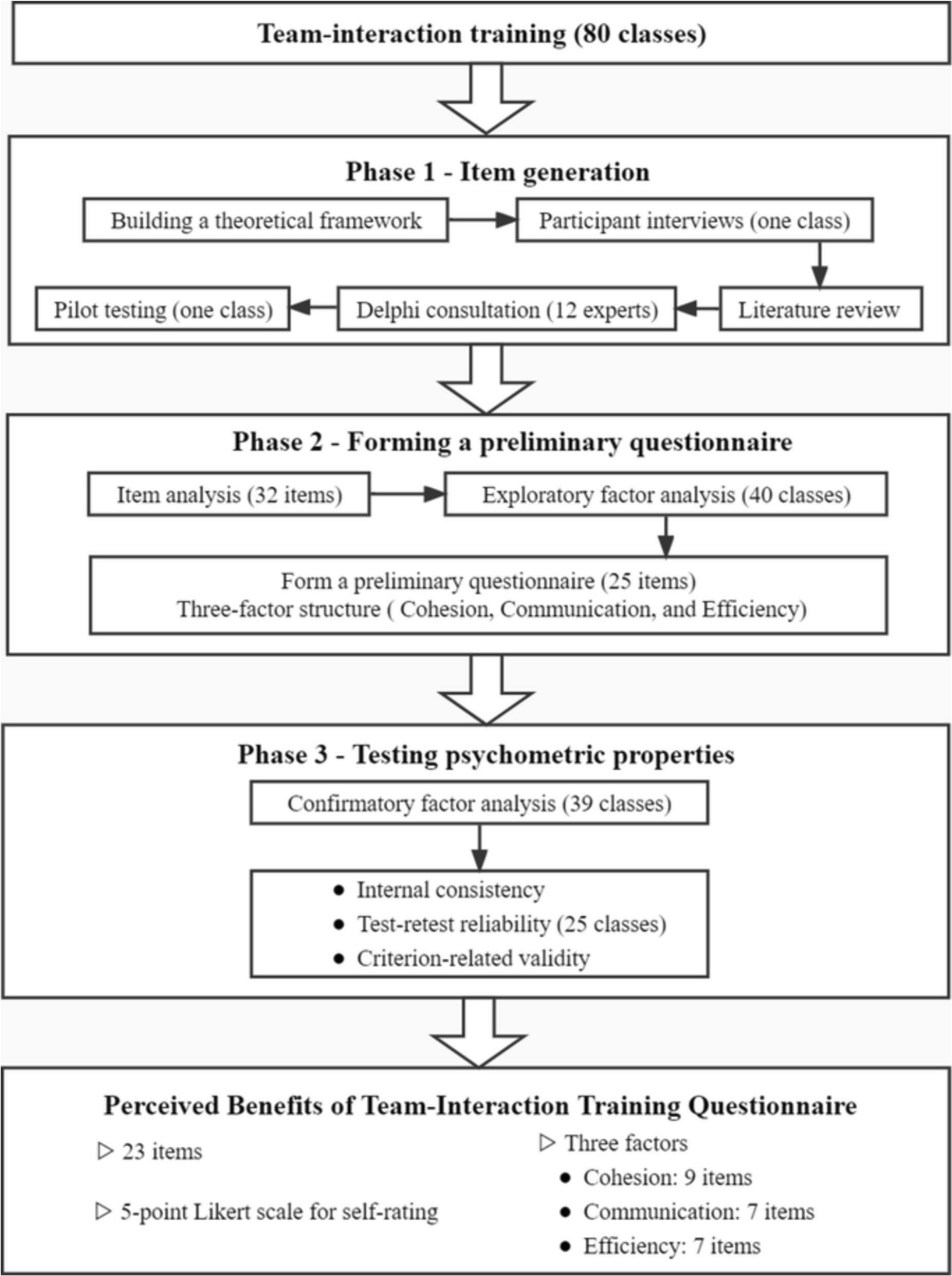
Questionnaire development and validation process

This study was conducted in three phases (Fig. 1). Phase 1 consisted of item generation: Items to generate the PBTITQ based on a theoretical framework of this study, participant interviews, a literature review, Delphi consultation, and pilot tests. Phase 2 consisted of forming a preliminary questionnaire: The preliminary questionnaire was formed through item analysis and exploratory factor analysis (EFA). Phase 3 consisted of testing psychometric properties: Psychometric characteristics of questionnaires were assessed using confirmatory factor analysis (CFA), internal consistency, test-retest reliability, and criterion-related validity.

### Design

From October 25 to December 31, 2021, team-interaction training was conducted among 1,698 first-year undergraduates in 80 classes at China Medical University. These participants were asked to complete the PBTITQ after five 90-minute sessions of team-interaction training. The basic demographic information of the participants is shown in Table 1. The training course schedule is shown in Table 2. This course was coordinated and arranged by three Physical Education teachers who had received training in psychological counselling. A teacher was responsible for explaining the rules before each practical activity and summarising the content of the course at the end to clarify any confusion of the participants. The other two teachers were responsible for cooperating in organizing the course activities. During the study, undergraduates provided informed consent, participated voluntarily and could withdraw at any time.

**Table 1:** General demographic information of the participants

Characteristics	N (%) or (Mean ± SD)
Mean Age	19.33 ± 0.65
Gender	
Male	568 (33.45)
Female	1130 (66.55)
Had similar team-interaction training experience or not	
Yes	775 (45.64)
No	923 (54.36)
Had group cooperation experience or not	
Yes	1601 (94.29)
No	97 (5.71)

**Table 2:** The team-interaction training lesson series

Sessions	Time allocation	Course type	Contents	Significance
Two sessions	45 min	Theory	Theoretical teaching: Teachers introduce the content and significance of the course and arouse the enthusiasm of students in this team activity.	The goals were to prepare to start the course and understand what it is about, to enhance students’ self-confidence, to overcome psychological inertia and persevere in challenges.
	45 min	Practice	Icebreaker: The session creates a positive team atmosphere and organizes team-interaction activities that encourage communication and interaction. For example, untying a bracelet: Members of each group stand in a concentric circle, first use their right hand to hold the hand of the person opposite them. They then use their left hand to hold the left hand of a nonadjacent person. Without letting go, they need to find a way to open this messy net.	The goals were to ① build harmonious interpersonal relationships between participants; ② correct participants’ prejudice against team-interaction and encourage them to treat the course with a positive attitude; and ③ establish a close relationship between the participants and the teacher. This also allowed the participants know and accept the teacher for this course.
	30 min	Practice	Team Building: The teacher divides all participants into teams. The captain, vice-captain, and flag bearer are identified for each team. The team names, team songs, and team slogan are determined together and shared.	The goals were to ① prepare mentally and physically for training so that participants get to know and understand each other; and ② encourage communication, narrow interpersonal distances, and assemble a preliminary unified team.
	60 min	Practice	From here, healthy competition is integrated into the curriculum. Jiaolong goes to sea: Teammates use leggings to link their feet in a row. They proceed sideways on a prescribed route to get from the starting point to the end in the shortest possible amount of time. In this project, adjusting their group rhythm and cooperating as a team is key to the team’s success.	The goals were to ① cultivate team-interaction and cooperation; ② establish a standardized team process; ③ improve organizational efficiency; and ④ understand the significance and important role of a unified command.
Two sessions	60 min	Practice	Quick Flop: The instructor puts 13 cards with numbers 1–13 on the table in any shape and asks the participants to take turns to flip the cards in order from 1 to 13. Cards need to be folded back into their original positions if the order of the turned cards does not match. The first team to turn over all the cards within an established time wins.	The goals were to ① let participants learn to encourage others; ② learn to dedicate and contribute; ③ enhance the ability of the team to determine an effective division of labour and overall planning; and ④ learn to use time reasonably.
	60 min	Practice	Haste 60 s: A circle with a radius of two metres has cards with numbers or images numbered 1–30. Each participating team selects a representative to collect cards in the area in order from 1 to 30. Other team members are only allowed to stand outside the circle and provide oral assistance to those inside. Each round of the challenge lasts no more than 60 s. The team with the most cards in the shortest amount of time wins.	The goals were to ① cultivate team members’ awareness of active communication and experience effective communication methods; ② clarify that empathy is the premise of communication; ③ clarify how the team is able to execute a plan; and ④ clarify each member’s position and importance in the team.
	60 min	Practice	Team building towers: Each group is given some materials and asked to build the prettiest tower in 25 min. The tower height should be at least 50 cm, with a reasonable structure and a beautiful appearance. After completion, each group presents their work and is evaluated by the teacher.	The goals were to ① Let team members be creative in executing team tasks and let each team member play their own role and contribute to the completion of team tasks; and ② Let members realize the importance of participation.
One session	90 min	Theory	Participant sharing and presentation: After the entire course, each participant shares their feelings related to team-interaction training. Finally, teachers make a summary of the whole course.	The goals were to make participants realize their own shortcomings and have a new understanding of the setbacks and problems in teamwork in this course, work, and life, to transform and improve their abilities.

### Three phases of questionnaire development

#### Phase 1- item generation

##### Building a theoretical framework

**Fig. 2:**
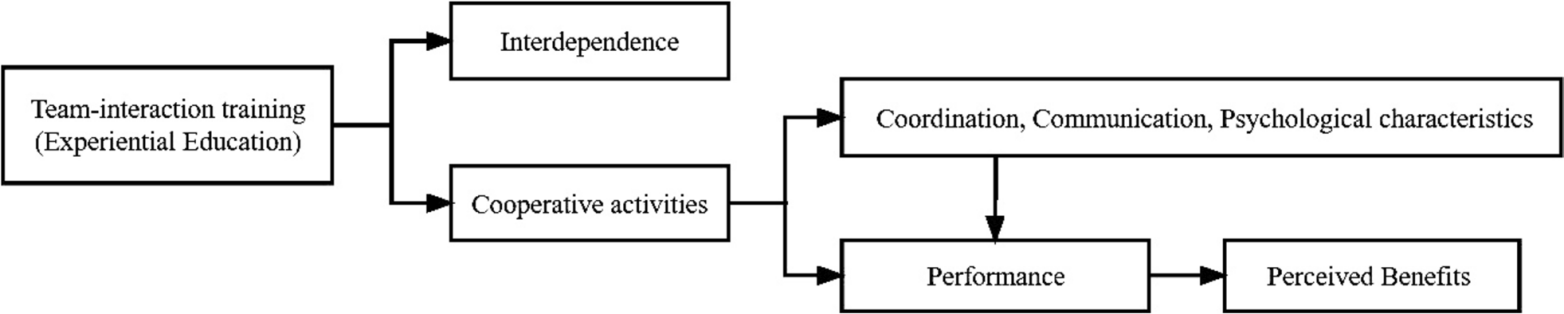
The theoretical framework for developing the Perceived Benefits of Team-Interaction Training Questionnaire

The theoretical framework of this study was based on social cognitive theory, self-efficacy theory, and sports performance models [15–17]. Social cognitive theory emphasizes that human cognition, environment and behaviour are not independent of each other but in a dynamic reciprocal relationship in which they constantly interact and influence each other [15]. Therefore, in this study, we defined perceived benefits of team interaction as an organically combined cyclical process rather than as a step-by-step process with independent components. Self-efficacy theory argues that successful experiences with activities help individuals achieve higher perseverance, better behavioural choices, and more positive emotional states [16]. From this perspective, we determined that team activities affect participant performance and perceived benefits. Sports performance models suggested that coordination, communication, and psychological characteristics all affect performance in sports [17]. Therefore, we considered these factors as influencing postcooperative performance. As a result of the above understanding, we constructed the theoretical framework of this study (Fig. 2).

##### Participant interviews and literature review

After the course, a class (*N* = 24) was randomly selected by lottery from 80 classes. Students were asked about their perceived benefits from team-interaction training. Additionally, we reviewed the literature on the perceived benefits of team-interaction training to construct questionnaire items more comprehensively.

A systematic search of several databases (e.g., Google Scholar, PubMed, ProQuest and Japan Medical Abstracts) was conducted, focusing on content about undergraduate students’ perceived benefits of team interaction as of October 1, 2022, searched through the following keywords: “team”, “interaction”, “teamwork”, “collaboration”, “questionnaire”, “training”, “physical”, “survey”, “undergraduate”, “benefits”, “Perception”, “Tools” and “Education”.

Finally, 52 items were developed and identified for this study based on the theoretical framework, literature review, and perceived-benefit interviews with participants.

##### Delphi consultation to study consensus on the items

We conducted a Delphi consultation to test the content of the initial questionnaire. A total of 12 experts were invited to participate. Their research areas included sports, pedagogy, psychology, and exercise psychology. The median and quartile of expert working years were 17.50 (14.25, 30.00). All experts had senior titles, including five professors and seven associate professors. Among them, eight had a master’s degree or above, and four had a bachelor’s degree. The importance of the items was assessed from 1 (very unimportant) to 5 (very important) using a Likert scale. Experts provided feedback and advice for item improvement.

We calculated the expert authority coefficient using the following formula: Cr = (Ca + Cs)/2 (Ca refers to the expert’s judgement criteria, and Cs refers to the level of familiarity with the items). To calculate the item-level content validity indices (CVI), we divided the number of experts who selected levels 4 (important) and 5 (extremely important) on a particular item by the total number of experts. Items with CVI < 0.80 were considered for modification or deletion [18]. In the second Delphi consultation, the same group of experts answered the questions and agreed on the evaluation of the questionnaire.

##### Pilot test

One class (*N* = 26) was randomly selected from 80 classes using a lottery. They served as participants in the pilot test using a Delphi postconsultation questionnaire. They were asked to assess the comprehensibility, readability, and completion time of the questionnaires. Based on these recommendations, the researchers made changes to the items’ wording. For reliability and validity, these data were excluded from the analysis.

#### Phase 2- forming a preliminary questionnaire

##### Item analysis

Items with the following situations were considered for deletion: critical ratio < 3, item-total correlation coefficient < 0.40, or factor loading < 0.45 [19].

##### Exploratory factor analysis

Exploring the latent factor structure of the questionnaire was done using EFA. The class that participated in the pilot test was not included in the candidates for EFA. Using class-based cluster sampling, 40 classes (*N* = 852) were randomly selected from the remaining 79 classes for EFA. The data of 59 participants were removed due to improper completion of the questionnaire. Finally, 793 effective response samples were collected, and the effective response rate was 93.08%.

Before factor analysis, we performed Bartlett’s test of sphericity and the Kaiser-Meyer-Olkin measure of factor validity to verify suitability for factor analysis. Maximum likelihood and promax rotation were used for EFA. Three criteria were used in the selection of the number of factors: (1) a scree-Test: the gravel plot showed a clear change between the steep slope of the large factor and the gradual tailing of its residual factor; (2) a Cronbach’s alpha of 0.70 or higher, and (3) the likelihood of factor interpretation [20]. At this point, the preliminary questionnaire was completed.

#### Phase 3- testing psychometric properties

##### Confirmatory factor analysis

In the CFA phase, we included the remaining 39 classes to test the effectiveness of the EFA-based measurement model. The data of 42 participants were removed due to irregular completion of the questionnaire. Finally, 778 effective response samples were collected, with an effective response rate of 94.88%. The preliminary questionnaire was used for CFA. The root mean square error of approximation, goodness-of-fit index, adjusted goodness-of-fit index, comparative fit index, and Tucker-Lewis index were used to assess the CFA goodness of fit [21].

##### Reliability: internal consistency and test-retest reliability analysis

The internal consistency of the questionnaire was tested using Cronbach’s alpha coefficient, and a value greater than 0.70 was considered acceptable. Test-retest reliability was used to consider stability. Two weeks after the questionnaire was completed, 25 classes (*N* = 601) were randomly selected in a lottery, and they were asked to complete the same questionnaire again for test-retest reliability analysis. In this instance, each student had a dedicated number to ensure that the same person did not complete the questionnaire twice and to easily match the retest questionnaire. This number was strictly confidential and not revealed to others.

##### Criterion-related validity

The Group Environment Questionnaire (GEQ) and Team Development Measure (TDM) were used to measure criterion-related validity. The GEQ was compiled by Carron et al. [11]. This study adopted the Chinese version of the GEQ revised by Ma in 2004 [22]. This questionnaire has been widely used to assess the team cohesion of undergraduate athletes, with Cronbach’s alpha coefficients exceeding 0.7 for all four subscales, indicating high reliability and validity [23]. It was scored on a 9-point scale, with a scale of 1, representing “strongly disagree”, to 9 representing “strongly agree”. It included four factors: Attraction to Individuals by Group Task, Attraction of Individuals by Group Interaction, Group Task Consistency, and Group Interaction Consistency. The GEQ contained 15 items, including two negative items. The negative items were reverse encoded. The Cronbach’s alpha for the GEQ in this study was 0.91.

TDM was compiled by Stock et al. [13]. The Chinese version of TDM used in this study was localized by Yan et al. in 2017 [24]. It has a Cronbach’s alpha of 0.68, which achieves reasonable reliability and good reliability in assessing teamwork [25]. It included four factors: Communication, Roles and Goals, Cohesion, and Team Primacy. TDM was scored using a 4-point Likert scale, with 1 to 4 representing “strongly disagree” to “strongly agree”. The TDM contained 28 items, including three negative items. The negative items were reverse encoded. The Cronbach’s alpha for TDM in this study was 0.93.

### Data collection

General demographic information and the PBTITQ were added to all questionnaires in this study. (1) General demographic information included age, gender, past experiences in team-interaction training and cooperation, and a dedicated number. (2) The PBTITQ was scored on a 5-point Likert scale, with 1 to 5 representing strongly disagree, disagree, generally agree, relatively agree, and strongly agree. In the CFA phase, we added the GEQ and TDM. All items of this questionnaire were in Mandarin. After team-interaction training, participants were given the questionnaires through the Questionnaire Star Platform, which is a professional software platform for online questionnaire design. Participants were asked to complete the questionnaire anonymously within 30 min and could only submit the questionnaire once without leaving blank items.

### Data analysis

Data were analysed using IBM SPSS 21.0 (*IBM*, Armonk, NY, USA) and AMOS 20.0 (*IBM*, Chicago, IL, USA). Descriptive analysis was performed on the characteristics of the participants. The negative items were reverse encoded. The Shapiro-Wilk test for normality and a Q-Q plot were used to perform tests for normality. If normality was not respected for at least one of these two tests, normality was rejected. The criterion-related validity of the PBTITQ was examined with Pearson correlation analysis. A two-sided *p* < 0.05 was used as the criterion for a statistically significant difference.

## Result

### Content validity

The values of Cr, Cs and Ca were 0.86, 0.79 and 0.92, respectively. In the first round of the Delphi consultation, the average CVI for the entire questionnaire was 0.87. We reviewed the 22 items to be revised one by one, deleted 11 items with a CVI lower than 0.8 and merged two items with similar meanings. In addition, three entries were removed because experts believed that they only applied to a small group of participants (e.g., As a leader, I have an obligation to lead teammates to victory). The remaining items were revised and entered into the second round of the Delphi consultation. Based on the first round of Delphi expert suggestions, we added two new items on cultural differences and listening to others. In the second round, the CVI for the items was between 0.89 and 1.00, and the average CVI was 0.94. The questionnaire reached consensus in the second round of the Delphi consultation, and a total of 39 items were formed.

### Item analysis

In the item analysis, it was found that critical ratio was < 3 for Items 3 and 17, the item-total correlation coefficient was < 0.40 for Items 10 and 30, and factor loadings were < 0.45 for Items 24, 26, and 38. By removing the above items, a questionnaire with 32 items was formed. The questionnaire was then used for EFA.

### Construct validity

The Kaiser-Meyer-Olkin coefficient was 0.876, and Bartlett’s test of sphericity yielded a significant value of < 0.01.

We examined the underlying construct of the 32 items by conducting EFA. Finally, three potential factors were determined, and the cumulative variance contribution rate was 82.60%. Items 9, 11, 16, 23, 25, 29, and 32 were excluded due to factor loadings < 0.50. Therefore, a total of 25 items in three factors entered CFA (Table 3).

In conjunction with the theoretical framework of this study, each factor was explained based on the three factors generated by EFA. Factor 1 is composed of ten items, called Cohesion. Factor 2 is composed of seven items and is named Communication. Factor 3 is composed of eight items and is named Efficiency. Overall correlation coefficients for the items range from 0.522 to 0.821, with all *p* < 0.05.

**Table 3:** Exploratory factor analysis of the Perceived Benefits of Team-Interaction Training Questionnaire

Items	Factor loadings		
	1	2	3
**Cohesion**			
36. I will trust my team members/team leader if I am a team leader/team member in the future.	0.825		
14. I’ve learned to respect others’ values after participating in team-interaction training.	0.788		
39. I believe in the ability of my teammates.	0.728		
15. I’ve learned to take the advice of others seriously after participating in team-interaction training.	0.707		
35. I can trust others in future group projects after participating in team-interaction training.	0.689		
22. I’ve learned how to work with team members after participating in team-interaction training.	0.688		
13. I appreciate the contributions of others after participating in team-interaction training.	0.670		
31. I think team members are obliged to lend a hand if a member is in trouble.	0.659		
28. I think unified command and unified action are not as efficient as individual actions.	0.633		
12. I believe that team members should encourage each other and make progress together.	0.620		
**Communication**			
6. I can express my opinions regarding my teammates’ decisions in an appropriate way.		0.921	
5. I will express my opinion frankly if there is a problem.		0.891	
7. I will encourage my teammates to express their ideas to improve communication within the team.		0.759	
4. I will provide positive feedback on others’ opinions.		0.668	
2. I am able to communicate with others more freely after participating in team-interaction training.		0.626	
3. I would prefer to resolve conflicts and problems in a communicative manner within a team.		0.535	
1. I’ve learned how to listen to the opinions and ideas of others after participating in team-interaction training.		0.522	
**Efficiency**			
37. I think we are more likely to win if I have confidence in the team.			0.817
34. I think a trusted team is more likely to win.			0.799
21. I think strong perseverance plays an essential role in sticking to the team’s common goal.			0.696
20. I can regulate my negative emotions when unexpected situations arise in team-interaction.			0.676
18. Strong-willed, emotionally stable members increase the team’s chances of winning.			0.596
27. Resource coordination within a team increases the chances of winning.			0.585
33. I cannot fully trust my teammates when doing tasks in a team.^a^			0.584
19. I will face my future challenges positively rather than run away from them.			0.581
Eigenvalue	19.236	1.167	1.077
Cumulative percentage of variance explained (%)	69.088	77.689	82.602

Back translation was conducted by two bilingual speakers of Chinese and English

^a^Negative item

### Construct validity

**Fig. 3:**
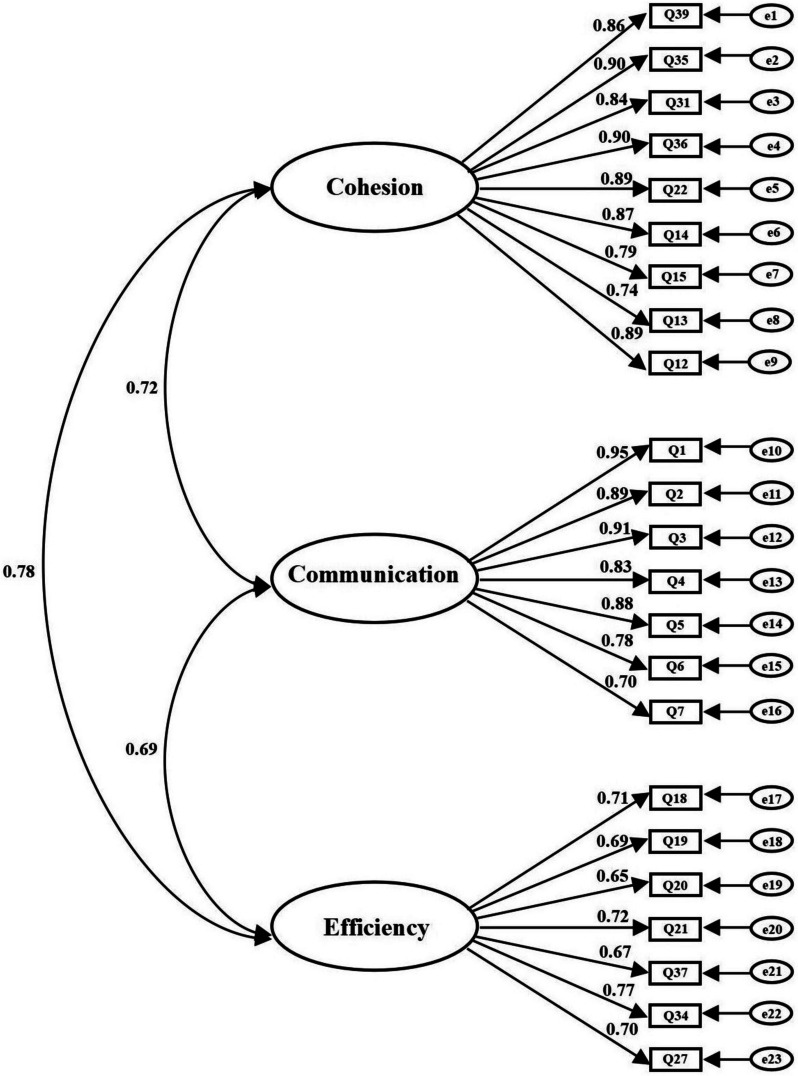
Confirmatory factor analysis of the Perceived Benefits of Team-Interaction Training Questionnaire

CFA tested the validity of the three-factor measurement model based on EFA. Items 28 and 33 were excluded due to low factor loading. A questionnaire with 23 items was formed at this stage (see Online Resource 1 for English version). The results showed that the model fit was good (χ^2^/df = 2.353, root mean square error of approximation = 0.068, goodness-of-fit index = 0.903, adjusted goodness-of-fit index = 0.922, comparative fit index = 0.925, Tucker-Lewis index = 0.918). Therefore, it was suggested that the questionnaire has reasonable construct validity (Fig. 3).

### Reliability

Cronbach’s alpha for the PBTITQ was 0.90. For factor reliability, the Cohesion coefficient was 0.89, the Communication coefficient was 0.92, and the Efficiency coefficient was 0.80. The test-retest reliability for the questionnaire was 0.88, and the test-retest reliability of the three factors were 0.78, 0.81, and 0.73. The split-half of the questionnaire was 0.81, and the split-half of the three factors were 0.70, 0.80, and 0.79.

### Criterion-related validity

The PBTITQ significantly correlated with GEQ (*r* = 0.808, *p* < 0.05) and TDM (*r* = 0.796, *p* < 0.05). The results showed that the PBTITQ had good criterion-related validity with both questionnaires (Table 4).

**Table 4:** The correlation coefficient between PBTITQ, GEQ, and TDM

Factors	PBTITQ			
	Cohesion	Communication	Efficiency	Total PBTITQ
Attraction to Individuals by Group Task	0.801^**^	0.666^**^	0.802^**^	0.803^**^
Attraction of Individuals by Group Interaction	0.792^**^	0.665^**^	0.730^**^	0.801^**^
Group Interaction Consistency	0.652^**^	0.603^**^	0.651^**^	0.656^**^
Group Task Consistency	0.748^**^	0.682^**^	0.856^**^	0.801^**^
Total GEQ	0.803^**^	0.697^**^	0.882^**^	0.808^**^
Communication	0.741^**^	0.654^**^	0.672^**^	0.793^**^
Roles and Goals	0.675^**^	0.629^**^	0.693^**^	0.717^**^
Cohesion	0.738^**^	0.709^**^	0.714^**^	0.793^**^
Team Primacy	0.634^**^	0.632^**^	0.603^**^	0.679^**^
Total TDM	0.784^**^	0.732^**^	0.725^**^	0.796^**^

*PBTITQ *Perceived Benefits of Team-Interaction Training Questionnaire, *GEQ *Group Environment Questionnaire, *TDM *Team Development Measure

^**^p < 0.01

## Discussion

This study developed and validated the PBTITQ among undergraduates based on a theoretical framework, participant interviews, and a literature review. The results showed that the PBTITQ has favourable psychometric properties, including reliability, validity, and good internal consistency among subscales. These results indicated that the PBTITQ was a valid and reliable tool for measuring undergraduates’ perceived benefits following team interaction.

The final PBTITQ consisted of 23 items with 5-point ratings. However, all five negative items (Items 8, 10, 17, 28, and 33) were excluded from the questionnaire. While it is recommended that negative items be included in questionnaires to reduce default bias, some experts advise against their use because they lead to inconsistent responses and are difficult to specify in the measurement model [26]. After excluding these and other inappropriate items, the measurement characteristics of the PBTITQ version were favorably improved.

The final version of the questionnaire included three factors, namely, Cohesion, Communication, and Efficiency. Based on the high correlation coefficients among the three factors, the overlap of differences between the factors suggests the existence of a common source of constructs [27], namely, perceived benefits of team-interaction.

The study also implied that the definitions of these three factors can be subsumed under the larger structure of perceived benefits from team interactions. This study provided sufficient evidence to demonstrate the validity of the PBTITQ with a three-factor construct. In a specific situation or game, the common goal of the group greatly increases the cohesion of the participants, who continually communicate and exchange opinions and finally improve their efficiency. Additionally, whether experiencing successes or failures, participants continue to accumulate experience, learn from, and better communicate with groups, ultimately improving team cooperation efficiency and achieving more success [28].

Cohesion, the first factor of the questionnaire, has been defined as a nonstatic process mainly driven by the tendency of a group to stick together and remain united in pursuit of instrumental goals and/or to satisfy participant affective desires [29]. A previous study pointed out that team cohesion, as an emerging emotional state, has an influential impact on both teams and individuals [30]. Cohesion was associated with positive mental health, reduced team conflict, and improved performance [31–33]. For example, Item 14, “I’ve learned to respect others’ values after participating in team interaction training,” reflects the benefit participants perceive from respecting teammates’ values. In teams, value differences are inevitable. Only by integrating values into the team, recognizing real differences in values, and balancing the relationship between various values, can the competitiveness of the team be enhanced, and team conflicts be reduced.

The Cohesion factor of our questionnaire mainly focuses on the participants’ perceived benefits after training from the aspects of trust, respect for teammates, cooperation and mutual assistance. Through this factor, the cohesion level of students can be accurately identified to help educators conduct targeted teaching and ultimately improve cohesion. High cohesion helps to build team structures, allowing participants to communicate openly and learn from each other to experience higher levels of performance [34].

Communication is one of the essential elements of progress in human social practice and work achievement, requiring speakers to align with their listeners [35]. We can maximize the goal of effective communication through talking, listening, and nonverbal communication [36]. Based on action theory, actions or activities greatly improve participants’ abilities and result in an overall improvement [37]. However, without appropriate communication skills to solve the problems that arise among the participants in the team-interaction process, the participants will feel more pressure and the efficiency of the team will be reduced [38]. Therefore, improving communication skills is an effective way to promote team interaction.

With the emphasis on communication skills in various industries, universities have paid increasing attention to the training of undergraduate communication ability [39]. Previous self-assessment teamwork tools for medical students also included communication as an imperative aspect [40]. In the Communication factor, our questionnaire mainly examines the participants’ perceived benefits of training on the aspects of expression, listening, and interactive feedback. For instance, Item 1, “I’ve learned how to listen to the opinions and ideas of others after participating in team interaction training,” and Item 5, “I will express my opinion frankly if there is a problem,” indicate the students’ perception of the benefits of listening and speaking to others after training. Team-interaction training effectively helps participants provide feedback and improve team cooperation by recalling effective means of communication used during their training.

Efficiency is the third factor of the PBTITQ, which affects participants’ fit with the team and affects the overall performance and subsequent behaviour of the team [41]. That is, the more efficient the team is, the easier it is to perform well, and the easier it is to maintain a positive mentality among the participants. A study on team questionnaire development also identified efficiency as an imperative factor to measure the effectiveness of teamwork [42]. Therefore, in this factor, content that can improve team efficiency or team success is included.

Within the factor of Efficiency, a few items, such as Item 37, “I think we are more likely to win if I have confidence in the team,” and Item 20, “I can regulate my negative emotions when unexpected situations arise in team-interaction,” revealed the participants’ perceived benefits of team confidence and self-regulation to overall team performance. By reviewing this content, participants can modify their behaviours to achieve high team performance or acquire a high level of awareness of team conditions through spontaneous adaptation to dynamic changes in the competitive environment [43]. This pattern of constantly making behavioural adjustments is not only the result of perceived benefits but also the way that this study proposes that individuals can improve team effectiveness.

### Strengths and limitation include implications for practice

The main advantage of this study is the innovative nature of the questionnaire. An extensive review of the literature was carried out prior to the preparation of the questionnaire. As of yet, there is no suitable questionnaire on the perceived benefits of team-interaction training for undergraduates. We emphasize the importance of teaching team-interaction in universities. Moreover, the items of the questionnaire were decided based on qualitative data collected from undergraduates involved in team interactions with a deep understanding of benefit perception. This allowed the properties of benefit perception to be accurately captured in team-interaction situations.

Despite these strengths, this study is subject to several limitations. This experiment was open only to first-year undergraduates, and the general applicability of the PBTITQ needs to be further validated when applied to other professional classifications or students in other years. In addition, this questionnaire was conducted in a medical university, and women accounted for the majority of the sample population. If the male-to-female ratio is changed, the findings may require further exploration. Finally, due to the limitations of the school curriculum, only one relatively common form of team interaction training was set up in this study, and other forms of training were not implemented in the process of questionnaire development. Therefore, the evaluation efficacy of the PBTITQ for other forms of team-interaction training needs to be further explored. It is suggested that training with a more representative population and team-interaction projects with different forms of content can be carried out in the future. This will further test the validity of the questionnaire.

University faculty can incorporate these skills into their course design or conduct extracurricular activities such as the experiential education used in this study [44, 45]. Such courses are designed to allow participants to actively learn and experience team-interaction in a realistic and concrete setting. Teaching such team-interaction at universities is critical, especially for medical students. In the healthcare field, a variety of problems arise between team members as a result of poor teamwork, which greatly increases the chances of harming patients [46]. A team-interaction course can significantly improve the individual and team skills of healthcare professionals entering the clinic, allowing them to rationalize the distribution of work and improve work efficiency [44, 47]. At the same time, it can also improve the quality of work and patient satisfaction. It prepares healthcare to enter into multidisciplinary collaboration and create a team-oriented professional healthcare environment.

## Conclusion

The PBTITQ reflected the three-factor structure formed by benefit perception in the context of team interaction: Cohesion, Communication, and Efficiency. It was developed with a sufficient degree of reliability and validity. Currently, the topic is not widely covered. We developed the first instrument that can measure the perceived benefits of team-interaction training for undergraduates, strengthening the evidence with the discussability of the topic. This study may serve as a basis for future research to help curriculum developers incorporate such training into university curricula and improve medical students’ team-interaction skills once they enter the clinic. Additional future research should continue to explore the perceived benefits of team-interaction and assess the perceived benefits from the perspectives of different schools, specialties, and training modalities, and increase the number of items assessed in the questionnaire. This will improve undergraduate students’ team-interaction skills and provide an adequate foundation for entering the workplace.

### Supplementary Information

**Additional file 1.**

## Data Availability

The data that support the findings of this study are available from the corresponding author, Shuang Zang, upon reasonable request.

## Abbreviations

PBTITQ: Perceived Benefits of Team-Interaction Training Questionnaire
EFA: exploratory factor analysis
CFA: confirmatory factor analysis
GEQ: Group Environment Questionnaire
TDM: Team Development Measure
CVI: content validity indices
