# Supplementary material for: Development and validation of the Perceived Benefits of Team-Interaction Training Questionnaire (PBTITQ) among undergraduates
Source: BMC Med Educ. 2023 Nov 7;23:840. doi: 10.1186/s12909-023-04810-3 (PMC10630993; doi:10.1186/s12909-023-04810-3)
Supplement: Supplementary file 1 — Additional file 1. [file 12909_2023_4810_MOESM1_ESM.docx]

**Online Resource 1**

**Perceived Benefits of Team-Interaction Training Questionnaire (English version)**

| **Perceived Benefits of Team-Interaction Training Questionnaire**  INSTRUCTIONS: The following questions are intended to assess how you feel about this team-interaction training. There are 23 items in this questionnaire, with 5 options for each statement. Please read each item carefully, and choose the most suitable option according to your participation in this team-interaction training, and tick “√” in the corresponding box. There is no good or bad answer, so try to tick the option that best represents your opinion. Please respond to ALL questions, thank you. | | | | | |
| --- | --- | --- | --- | --- | --- |
| Item | Strongly agree | Relatively agree | Generally agree | Disagree | Strongly disagree |
| **Cohesion** | | | | | |
| 1. I will trust my team members/team leader if I am a team leader/team member in the future. | □ | □ | □ | □ | □ |
| 2. I’ve learned to respect others’ values after participating in team-interaction training. | □ | □ | □ | □ | □ |
| 3. I believe in the ability of my teammates. | □ | □ | □ | □ | □ |
| 4. I’ve learned to take the advice of others seriously after participating in team-interaction training. | □ | □ | □ | □ | □ |
| 5. I can trust others in future group projects after participating in team-interaction training. | □ | □ | □ | □ | □ |
| 1. I’ve learned how to work with team members after participating in team-interaction training. | □ | □ | □ | □ | □ |
| 7. I appreciate the contributions of others after participating in team-interaction training. | □ | □ | □ | □ | □ |
| 8. I think team members are obliged to lend a hand if a member is in trouble. | □ | □ | □ | □ | □ |
| 9. I believe that team members should encourage each other and make progress together. | □ | □ | □ | □ | □ |
| **Communication** | | | | | |
| 10. I can express my opinions regarding my teammates’ decisions in an appropriate way. | □ | □ | □ | □ | □ |
| 11. I will express my opinion frankly if there is a problem. | □ | □ | □ | □ | □ |
| 12. I will encourage my teammates to express their ideas to improve communication within the team. | □ | □ | □ | □ | □ |
| 13. I will provide positive feedback on others’ opinions. | □ | □ | □ | □ | □ |
| 14. I am able to communicate with others more freely after participating in team-interaction training. | □ | □ | □ | □ | □ |
| 15. I would prefer to resolve conflicts and problems in a communicative manner within a team. | □ | □ | □ | □ | □ |
| 16. I’ve learned how to listen to the opinions and ideas of others after participating in team-interaction training. | □ | □ | □ | □ | □ |
| **Efficiency** | | | | | |
| 17. I think we are more likely to win if I have confidence in the team. | □ | □ | □ | □ | □ |
| 18. I think a trusted team is more likely to win. | □ | □ | □ | □ | □ |
| 19. I think strong perseverance plays an essential role in sticking to the team’s common goal. | □ | □ | □ | □ | □ |
| 20. I can regulate my negative emotions when unexpected situations arise in team interactions. | □ | □ | □ | □ | □ |
| 21. Strong-willed, emotionally stable members increase the team’s chances of winning. | □ | □ | □ | □ | □ |
| 22. Resource coordination within a team increases the chances of winning. | □ | □ | □ | □ | □ |
| 23. I will face my future challenges positively rather than run away from them. | □ | □ | □ | □ | □ |

Likert scale: Strongly agree = 5; Relatively agree = 4; Generally agree = 3; Disagree = 2; Strongly disagree = 1
